# Carbonyl emissions from heated tobacco products

**DOI:** 10.18332/tpc/214783

**Published:** 2026-02-18

**Authors:** Efthimios Zervas, Niki Matsouki, Charikleia Tsipa, Zoe Gareiou

**Affiliations:** 1 School of Applied Sciences and Sustainable Design, Hellenic Open University, Patra, Greece

**Keywords:** formaldehyde, emissions, acetaldehyde, carbonyls, propionaldehyde

## Abstract

**INTRODUCTION:**

The scope of this work is to determine the carbonyl emissions from five heated tobacco products (HTPs) and the existence of differences in the emissions based on the brand, on the stick (having a different flavor) and on the puffing regime.

**METHODS:**

Carbonyls were determined in the mainstream emissions of HTPs. Vapors from 5 commercial HTPs: IQOS, LIL, PULZE, ILUMA and GLO, and fifteen different stick flavors were generated using a peristaltic pump under both ISO and Canadian puffing regimes. Carbonyls were collected in an impinger containing a 2,4-dinitrophenylhydrazine (2,4-DNPH) solution and analyzed using HPLC-UV.

**RESULTS:**

All HTPs emit carbonyls. Acetaldehyde, propionaldehyde and butyraldehyde are detected in quantities varying from 34.35 to 72.11 μg/stick, 3.28 to 8.58 μg/stick and 3.07 to 6.20 μg/stick respectively, for the different brands and stick flavors. Formaldehyde and crotonaldehyde are found below the detection limit of the analytical method. Acetone and acrolein co-eluted and cannot be quantified. Under ISO regime, ILUMA emits more acetaldehyde than GLO, more propionaldehyde compared to the other brands and more butyraldehyde compared to PULZE, LIL and GLO. Under Canadian regime, no significant variations were found between the brands, except IQOS that emits more acetaldehyde than GLO. Also, the difference of the sticks, thus having a different flavor, within the same brand, has an impact on the emissions.

**CONCLUSIONS:**

Although HTPs are promoted as products with reduced risk compared to conventional cigarettes, still the detection of carbonyls in HTPs emissions is a fact and needs further research. All five HTPs used here and all sticks used emit carbonyls. Carbonyls’ concentrations are found to vary significantly among the different brands, the difference of the sticks, and concerning flavor, and to increase when changing to a more intense puffing regime.

## INTRODUCTION

Smoking of conventional cigarettes (CCs) is known to have significant negative impacts on human health^1^. Heated tobacco products (HTPs), are a relatively recent addition to the tobacco market and are promoted by the tobacco industry as having lower health impacts compared to CCs^2^. HTP manufactures claim that these products heat rather than burn tobacco^3^, limiting in this way the toxic products of combustion, typically found in CC smoke^4^. However, some researchers^5^ claim that combustion still occurs in HTPs and a number of toxic compounds such as carbonyls, nitrosamines, polycyclic aromatic hydrocarbons, particles etc., have been detected in their mainstream emissions. Although studies generally report that these emissions are found in lower concentrations in the case of HTPs than in the case of conventional cigarettes^6-8^, not all researchers confirm this statement^9^. Many of the above chemical compounds are known to have severe negative health effects, e.g. carbonyls can be carcinogens, respiratory and cardiovascular toxicants, and even addictive^10^. Moreover, the novelty of these products has not yet allowed for full knowledge understanding of their long-term health impacts^9,11^. In addition, the increasing popularity of HTPs^12^, especially among adolescents and the young age of many users^13^, are additional factors that must be considered when evaluating the safety of these products^14^.

A large part of the scientific data available for the safety assessment of HTPs has been produced from Philip Morris International (PMI), the company that introduced the novel tobacco product under the name IQOS in 2014^4,9^. Since then, several other manufacturers have entered the market, such as British American Tobacco (BAT), KT&G, Imperial Brands or Japan Tobacco International (JTI), producing devices under the brand names GLO, LIL SOLID, PULZE and Ploon Tech, respectively. PMI has also recently launched a new product called ILUMA. Α wide variety of different heated tobacco sticks, having a different flavor are currently available on the market, which can be used with similar – though not identical –devices.

It is well established that heating temperature is a key factor influencing the chemical composition of emissions from heated or combusted materials^15^. We hypothesize that differences in heating temperatures contribute to variations in emissions between brands, since each operates at a different temperature. According to manufacturers, the maximum heating temperature is 350^o^C for IQOS, ILUMA and LIL, 345^o^C for PULZE and 280^o^C for GLO. Furthermore, different heating technologies are employed, since IQOS, LIL and PULZE heat the tobacco from the center using a flat blade, a needle blade, and a cylindrical ceramic rod, respectively, whereas ILUMA and GLO heat the tobacco from the perimeter. The chemical composition of the emissions also depends on the stick substrate composition^16,17^, which varies across different brands.

Carbonyls are a group of compounds that are classified as hazardous or potentially hazardous compounds (HPHCs) and are detected in the emissions of all tobacco products, including HTPs. Previous studies have confirmed the presence of carbonyls in HTPs emissions, with these compounds being among the most abundant HPHCs found in the aerosol^18^. However, a standardized method for carbonyl determination in HTP emissions has not yet been established. Existing studies differ in key aspects such as sample generation, carbonyl collection methods, and analytical techniques. This methodological variability leads to significant discrepancies in reported carbonyl concentrations, making cross-study comparisons unreliable. For instance, acetaldehyde has been reported at 35.48 μg/item using Neosticks from BAT, puffed under Canadian regime^19^. In another study, levels ranged from 0.17 to 27.6 μg/item when the heating temperature varied between <250^o^C and 320^o^C, using a new cigarette smoke generating (SNCSG) system based on heating-temperature control, with DNPH-cartridge and analyzed with HPLC^20^. Meanwhile, for IQOS under five different puffing regimes, acetaldehyde levels ranged from 156.67 to 198.59 μg/item using 2,4-DNPH-coated silica cartridges and HPLC analysis^21^.

Additionally, the impact of flavor additives in the tobacco sticks on carbonyl emissions remains largely unknown. Given that various flavors are used in these products, further research is necessary to determine how flavoring agents influence emission profiles. The aim of this work is to determine carbonyls emissions, under the same experimental conditions for different HTPs brands, using several different sticks having different flavors under two different puffing regimes (ISO and Canadian) and to compare the emissions between the brands and different sticks that have different flavors.

## METHODS

### Devices and sticks

Five types of commercial HTPs were used in this work: IQOS (Originals Duo), IQOS ILUMA and LIL (Philip Morris), PULZE (Imperial Tobacco) and GLO (British American Tobacco). Different sticks having three different tobacco flavors per device, 15 different sticks in total, are tested: Yellow Selection (YS), Silver Selection (SS) and Turquoise Selection (TS) for IQOS and ILUMA; Regular (R), Roxo (ROX) and Marine (M) for LIL; Capsule Polar (CP), Ice (I) and Rich Bronze (RBr) for PULZE, and Classic Tobacco (CT), Arctic Click (AC) and Scarlet Click (SC) for GLO. It should be mentioned that the flavored sticks used with IQOS and ILUMA are different, though they have the same name. The sticks’ physical characteristics per brand (weight, diameter and length) are presented in the Supplementary file.

### Emissions generation

Mainstream smoke was generated using a peristaltic pump (Masterflex L/S 07522-20, Cole-Palmer) connected to the HTP device. The device was operated under both ISO and Canadian puffing regimes. For the ISO regime, the pump flow rate was set to 35 mL per puff with a puff interval of 60 s. For the Canadian regime, the pump flow rate was set to 55 mL per puff with a puff interval of 30 s. In both cases, the puff duration was 2 s. Each stick was used smoked until the device automatically switched off. The number of puffs per stick varied depending on the device and puffing regime.

Each experimental data point was generated from the emissions of ten sticks of the same flavor, used consecutively. For each device, three experimental data points were collected for each of three different flavors, under both puffing regimes. Three experimental points per different stick of different flavor and three different flavors per device were performed under both regimes.

The repeatability of the method was assessed by performing four experimental data points (4×10 sticks), under identical conditions (ISO regime), using one stick type and flavor with a single device (ILUMA- TS).

### Emissions collection

The emissions generated were collected in an impinger containing 20 mL of 2,4-dinitrophenylhydrazine (DNPH) solution, kept at 4^o^C, for the derivatization of the carbonyls. A 2,4-DNPH solution was prepared by diluting 0.04 g of 2,4-DNPH in 100 mL of acetonitrile, containing 1 mL of H_2_SO_4_ (95–97%). The final solution remained for at least 30 min at room temperature to complete the derivatization reaction before been analyzed.

The effectiveness of one 2,4-DNPH impinger as a collection method was evaluated by performing one experiment (IQOS-S, ISO regime) using two impingers connected in series and by quantifying the compounds detected in each one of the two impingers.

### Analysis of HTPs emissions

The derivatized carbonyl compounds were analyzed using a HPLC-UV (Perkin-Elmer). Qualitative and quantitative identification was performed using an external standard (T0-11/IP-6A Aldehyde/Ketone-DNPH Mix, Supelco, USA). The separation column was Brownlee Choice C18 (150×4.6 nm, ID 5 μm), and the mobile phase was acetonitrile/water, HPLC grade, 60/40, v/v. The column temperature was 60°C, the flow rate was set to 1.0 mL/min and the injection volume was 20 μL. The detection wavelength was 360 nm.

### Statistical analysis

In order to investigate whether the detected carbonyls differ significantly between the two different puffing regimes, a t-test statistical analysis was applied. To investigate whether there is a statistically significant difference in carbonyls emission between different brands, a one-way ANOVA statistical analysis was applied for both puffing regimes: ISO and Canadian. One-way ANOVA statistical analysis was also be applied in order to investigate the existence of any statistical difference in the emissions depending on the different flavor among the same brand. The results of the t-test and the one-way ANOVA tests are presented in the Supplementary file.

## RESULTS

Under the experimental conditions, seven carbonyls were identified in HTP emissions. Formaldehyde and crotonaldehyde were below the detection limit of the analytical method. The other five, acetaldehyde, propionaldehyde, acetone, acrolein and butyraldehyde were detected at measurable concentrations; however, acetone and acrolein could not be quantified separately due to co-elution. For all brands and sticks having different flavors under both regimes, the average value in μg per stick of the acetone and acrolein mixture was approximately 3.5 times lower than the amount of acetaldehyde emitted per stick and about twice the amount of propionaldehyde. More specifically, the average quantity of the acrolein and acetone mixture was 14.68 ± 8.39 μg/stick.

A single impinger was sufficient to collect the majority of carbonyls, as the concentration in the second impinger was found to be <5% of that in the first. In terms of repeatability, when ILUMA, with flavor TS, was used under the ISO puffing regime, the mean quantities of acetaldehyde, propionaldehyde and butyraldehyde were 66.00 ± 3.07, 6.77 ± 0.41 and 5.33 ± 0.23 μg/stick, respectively. These values correspond to a relative standard deviation of 17.2%, 11.6% and 6.8%, respectively.

### Acetaldehyde emissions

**Figure 1:**
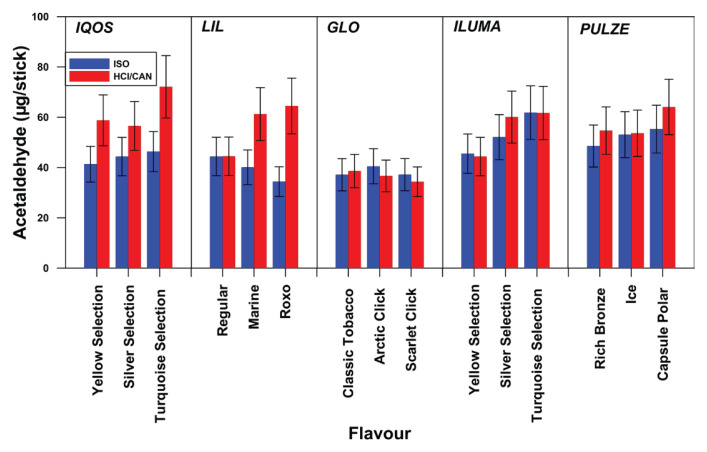
Acetaldehyde emissions (μg/item) per device and per flavor for ISO and Canadian puffing regime

Figure 1 shows acetaldehyde’s quantity for all sticks of different flavor tested per device, both for ISO and Canadian puffing regime. In Figure 1, the emission of acetaldehyde per brand/flavor is shown (mean value and standard deviation). The corresponding values per device are 41.32–46.34 μg/stick (ISO) and 56.53–72.11 μg/stick (Canadian) for IQOS, 34.40–44.41 μg/stick (ISO) and 44.49–64.47 μg/stick (Canadian) for LIL, 48.56–55.29 μg/stick (ISO) and 53.63–64.08 μg/stick (Canadian) for PUZLE, 45.51–61.86 μg/stick (ISO) and 44.39–61.69 μg/stick (Canadian) for ILUMA, and finally 37.14–40.51 μg/stick (ISO) and 34.35–38.60 μg/stick (Canadian) for GLO.

### Propionaldehyde emissions

**Figure 2:**
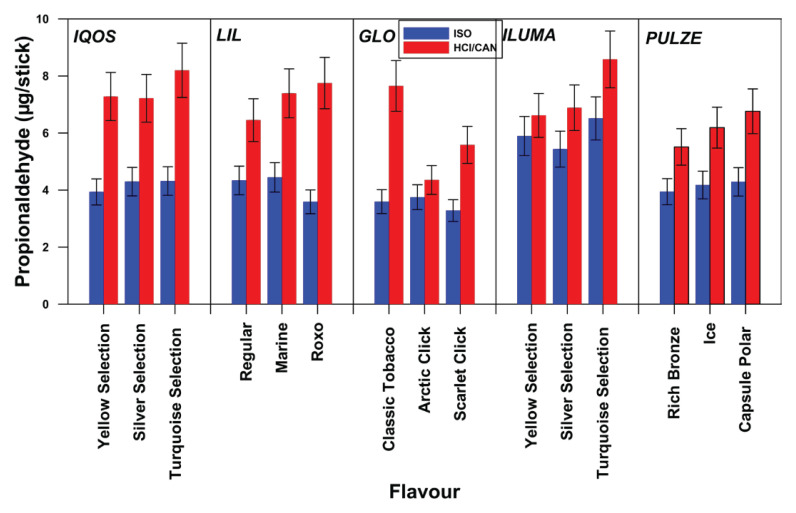
Propionaldehyde emissions (μg/item) per device and per flavor for ISO and Canadian puffing regime

Figure 2 shows propionaldehyde’s quantity of all sticks of different flavor tested per device, both for ISO and Canadian puffing regime. In Figure 2, the emission of propionaldehyde per brand/flavor is shown (mean value and standard deviation). The corresponding values per brand are 3.94–4.32 μg/stick (ISO) and 7.22–8.20 μg/stick (Canadian) for IQOS, 3.59–4.45 μg/stick (ISO) and 6.45–7.75 μg/stick (Canadian) for LIL, 3.95-4.29 μg/stick (ISO) and 5.51–6.76 μg/stick (Canadian) for PUZLE, 5.44–6.51 μg/stick (ISO) and 6.62–8.58 μg/stick (Canadian) for ILUMA, and finally 3.28–3.75 μg/stick (ISO) and 4.36–7.65 μg/ stick (Canadian) for GLO.

### Butyraldehyde emissions

**Figure 3:**
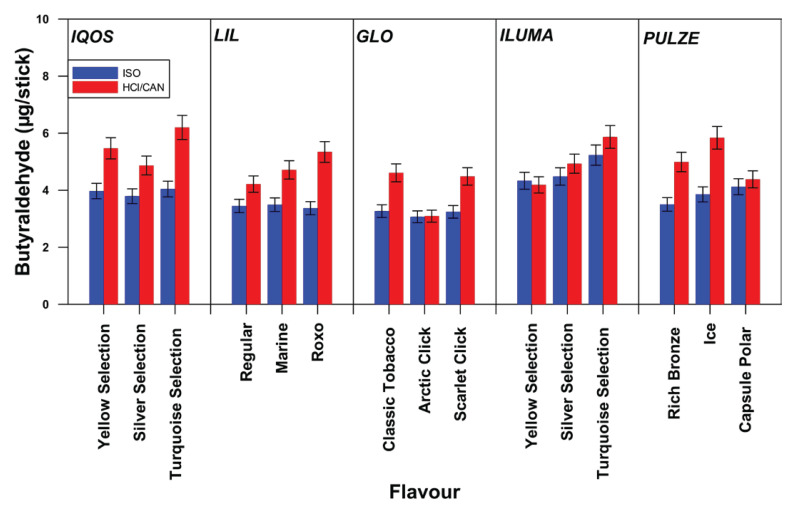
Butyraldehyde emissions (μg/item) per device and per flavor for ISO and Canadian puffing regime

Figure 3 shows butyraldehyde’s quantity for all sticks of different flavor tested per device, both for ISO and Canadian puffing regime. In Figure 3, the emission of butyraldehyde per brand/flavor is shown (mean value and standard deviation). The corresponding values per brand are 3.79–4.04 μg/stick (ISO) and 4.87–6.20 μg/stick (Canadian) for IQOS, 3.37–3.50 μg/stick (ISO) and 4.225.34 μg/stick (Canadian) for LIL, 3.51–4.12 μg/stick (ISO) and 4.38–5.84 μg/stick (Canadian) for PUZLE, 4.33–5.23 μg/stick (ISO) and 4.19–5.87 μg/stick (Canadian) for ILUMA, and finally 3.07–3.27 μg/stick (ISO) and 3.09–4.61 μg/stick (Canadian) for GLO.

### Comparison of emissions between smoking regimes, brands and flavors

The quantities of all three compounds were higher when Canadian puffing regime was used compared to the ISO regime and this difference was statistically significant. The results of the emissions comparison between different brands under both puffing regimes are shown in Table 1. Table 1 provides a visual summary of all the results obtained from the statistical analysis and is based on the data presented in Supplementary file Tables 2–10. As shown in Table 1, under the ISO puffing regime, ILUMA emitted a statistically significant higher amount of acetaldehyde than GLO, a higher amount of propionaldehyde compared to IQOS, PULZE, LIL and GLO and a higher amount of butyraldehyde compared to PULZE, LIL and GLO. Under the Canadian puffing regime, IQOS emitted a statistically significant higher amount of acetaldehyde compared to GLO, while no statistically significant differences were found between the brands for propionaldehyde and butyraldehyde.

**Table 1:** Carbonyls emissions comparison between different brands for both puffing regimes

*Compound*	*ISO*	*Canadian*
**Acetaldehyde**	ILUMA>GLO	IQOS>GLO
**Propionaldehyde**	ILUMA>IQOS, PULZE, LIL, GLO	No statistically significant difference between brands
**Butyraldehyde**	ILUMA>PULZE, LIL, GLO	No statistically significant difference between brands

**Figure 4:**
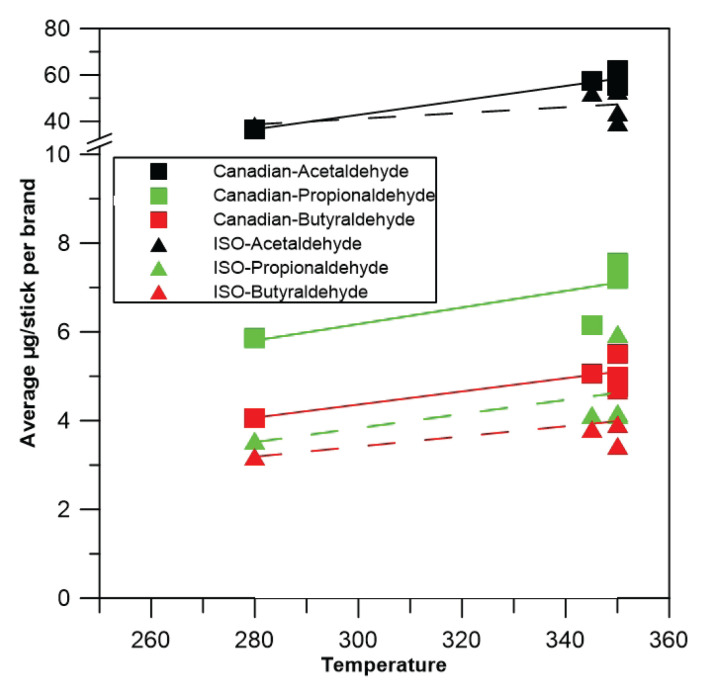
Carbonyls emissions versus heating temperature

Figures 1–3 illustrate significant discrepancies in the emitted quantities of carbonyls in relation to the stick type and its flavor; this discrepancy is more pronounced under the Canadian puffing regime, than under the ISO regime. One-way ANOVA statistical analysis confirmed these findings. Specifically, under the Canadian regime, a statistically significant difference was observed in the emissions of all carbonyl compounds, across different sticks of different flavor and brands, with the exception of acetaldehyde in the case of GLO. In contrast, under the ISO puffing regime some sticks of different flavors within brands showed statistically significant differences in carbonyl emissions, while others did not. More specifically, acetaldehyde emissions per different stick of different flavor differed significantly only for IQOS and GLO; propionaldehyde emissions differed only for IQOS and PULZE; and butyraldehyde emissions differed only for IQOS, LIL, and GLO. Regarding the emitted concentrations of carbonyls in relation to the device’s maximum heating temperature, Figure 4 shows that the quantities of emitted carbonyls slightly increase with temperature.

## DISCUSSION

As known, HTP devices are available in the market in a variety of different devices. Carbonyls have been detected in HTPs emissions in different quantities in previous studies, due to differences in the device and the different sticks of different flavor used, the vapors generation method, the collection method etc.^19,22,23^. This work analyzed the difference in carbonyls emissions under the same experimental conditions, using five devices of different brands and three different sticks of different flavor per brand and under two puffing regimes (ISO and Canadian). The devices differ from one another in a number of parameters, such as the maximum heating temperature, the heating technology, the composition of tobacco and flavor of the sticks, the materials used for the device manufacture etc. The present work, examines the impact of the device type, the difference of the sticks having different flavor and the puffing regime on carbonyls’ emissions.

Seven carbonyls were detected, and three, acetaldehyde, propionaldehyde and butyraldehyde, were quantified in the emissions of the 5 different brands of HTPs used with the 15 different sticks of different flavor, under both ISO and Canadian puffing regime. Formaldehyde and crotonaldehyde were below the detection limit and acrolein co-eluted with acetone, but the quantity of the mixture was on average 3.5 times less than that of acetaldehyde and double of propionaldehyde. Statistical analysis of the differences in carbonyls emissions in correlation to the puffing regime, showed that all the compounds had statistically significant higher concentrations when the regime was more intense (CAN comparing to ISO). This finding confirms the influence of the use parameters on the emissions, as in the case of conventional or electronic cigarettes^24,25^. When puff volume is set to 55 mL (Canadian regime) instead of 35 mL (ISO regime), the quantity of vapors collected per sample is higher, so it is expected to have higher amounts of carbonyls. Additionally, a short puff interval in the use of the devices under continuously high temperatures, is known to be a parameter correlated positively to the emissions^26^. Further analysis showed that acetaldehyde emissions differ significantly between the different brands and that brand ILUMA emits more acetaldehyde than GLO under ISO puffing regime, while IQOS emits more than GLO under the Canadian regime. ILUMA and IQOS, though based on different heating technologies, emit both higher quantity of acetaldehyde, compared to the other brands. Based on this, we assume that heating technology is not expected to be the main parameter affecting emissions, in agreement to our previous work concerning the emissions of particles^26^, but rather the heating temperature. Acetaldehyde production seems to depend on the device maximum heating temperature, which has the highest value for IQOS and ILUMA (350^o^C). Moreover, IQOS and ILUMA are manufactured by the same producer, so we suggest that other parameters correlating to the manufacturing characteristics, such as the materials of the device or the composition of the sticks may correlate with the emissions, and should be examined in future work. Propionaldehyde and butyraldehyde under the ISO regime, were respectively emitted in significantly higher quantities in the case of ILUMA compared to all the other brands and, in comparison, to PULZE, LIL and GLO. Under the Canadian regime, no significant differences were found for propionaldehyde and butyraldehyde between the brands. The lack of significant difference between brands under the Canadian regime confirms once again the correlation of emissions with temperature^26^. The difference of sticks having different flavor is also a parameter that affects the emissions of carbonyls; however, further experiments and analysis of the stick is considered necessary in order to assess which of the flavorings added during the stick manufacturing process increases those emissions. The differences between different sticks of different flavor among the same brands were found to increase with the increase in the emitted quantity of carbonyls, as was recorded during the Canadian puffing regime. Despite the lack of homogeneity, based on the use of different brands, sticks etc., the level of acetaldehyde, which was found to be the most abundant carbonyl compound, did not exceed 72.11 μg/stick. This value is significantly lower compared to literature data for conventional cigarettes, where values can reach up to 1605.8 μg/cig^27^ or up to 1616.56 μg/cig^28^. However, acetaldehyde’s presence in the emissions underlines that further research concerning HTPs’ health impacts is necessary. Though the quantity of carbonyls in HTPs emissions is lower than in CCs, carbonyls are crucial for public health, and policy makers should take their presence in HTPs emissions into consideration in future studies or policies related to these products.

### Limitations

The emissions shown here are valid under the experimental conditions used and cannot be extrapolated to other conditions. Additionally, a correlation of the emissions with the physical characteristics of the sticks, including reconstituted tobacco weight and height/diameter of the rod has to be performed. Optimization of the experimental conditions has to be performed in order to quantify the emissions of formaldehyde and crotonaldehyde and to perform a satisfactory separation of the chromatograph peaks attributed to acetone and acrolein. Moreover, since only 15 products were examined, an extension of this research, to include all the different sticks of different flavor of each brand, should be performed in order to explore the effect of stick composition and to confirm our findings about the effects of the device maximum heating temperature and heating technology on carbonyls emissions. However, though maximum heating temperature of the devices is known, a measurement of the tobacco material temperature during the heating process should also be performed. Also, Ploom Tech, and other hybrid products, are not studied here. Finally, when experimental data are used for health safety evaluation, it should always be taken into account that ISO and Health Canada protocols are not replicating human behavior.

## CONCLUSIONS

This study focuses on carbonyls emissions during HTPs use. For this purpose, five different devices with 15 different sticks of different flavor were purchased. Using a peristaltic pump for aerosol generation and 2,4-DNPH impinger for the collection of HTPs emissions, each experimental point, consisting of the aerosol produced after the heating of ten sticks, was analyzed for the detection of carbonyls. Three compounds (acetaldehyde, propionaldehyde and butyraldehyde) were found in quantities depending on the device and the puffing regime; formaldehyde and crotonaldehyde were below the method detection limit, while acetone and acrolein eluted at the same retention time and could not be quantified. Carbonyl’s quantity varies significantly between different brands, and heating temperature of the stick seems to be a dominant parameter for this variation. Increase of the heating temperature results in an increase in the emission of carbonyls. Concerning acetaldehyde, ILUMA and IQOS emit more than GLO, under ISO and Canadian conditions, respectively. ILUMA emits a significantly higher amount of propionaldehyde compared to all other brands and of butyraldehyde compared to LIL, PULZE and GLO, under ISO conditions. Under Canadian conditions, no difference was found in the emission of propionaldehyde and butyraldehyde among the brands, though carbonyl emissions are in general significantly increased when changing from ISO to Canadian puffing regimes, despite the brand. This finding may be attributed to the continuous high working temperature for all the devices due to the short puff interval. Further analysis of this trend, in terms of brand, showed that ineffective cooling of the device between puffs probably depends on the device heating technology. ILUMA and GLO, based on the same heating technology, do not have statistically significant increase in the emissions of carbonyls when changing from ISO to Canadian puffing regimes, in comparison to IQOS, LIL and PULZE. The different sticks of different flavor emitted statistically significant quantities of carbonyls, mainly under the Canadian puffing regime. Under the experimental conditions, the maximum concentration of the most abundant carbonyl compound was significantly lower than that found in the emissions of conventional cigarettes, suggesting reduced risk concerning this family of chemical compounds. However, further research in terms of safety is considered necessary.

## Supplementary Material



## Data Availability

The data supporting this research can be found in the Supplementary file.
