# Supplementary material for: Carbonyl emissions from heated tobacco products
Source: Tob Prev Cessat. 2026 Feb 18;12:10.18332/tpc/214783. doi: 10.18332/tpc/214783 (PMC12914568; doi:10.18332/tpc/214783)
Supplement: Supplementary file 1 [file TPC-12-11-s1.pdf]

Table 1. Physical characteristics of the sticks

Device	Weight (g)	Diameter (mm)	Length (mm)
**IQOS**	0.7532	7.26	45.30
**IQOS ILUMA**	0.6811	7.26	45.30
**LIL**	0.7482	7.23	45.40
**PULZE**	0.6198	6.97	48.27
**GLO**	0.6548	6.44	74.84

The table above presents the physical characteristics of the sticks, however, no correlation between size and emissions can be performed since the exact mass of the reconstructed tobacco for each stick is unknown. The measurements were conducted in our lab using an analytical balance (Kern-ADB, precision ± 0.0001 g) and a digital caliper (Lux Tools Comfort, precision ± 0.03 mm). The measurements of the dimensions do not have high accuracy because the sticks are compressible and there is variability of the force applied during measurements.

Table 2. Comparison between different brands - One-way ANOVA

**ANOVA**						
		**Sum of Squares**	**df**	**Mean Square**	**F**	**Sig.**
Acetaldehyde_ISO	Between Groups	581.150	4	145.287	6.330	0.008
	Within Groups	229.518	10	22.952		
	Total	810.668	14			
Acetaldehyde_Canadian	Between Groups	1193.302	4	298.325	4.736	0.021
	Within Groups	629.853	10	62.985		
	Total	1823.155	14			
Propionaldehyde_ISO	Between Groups	9.965	4	2.491	19.347	0.000
	Within Groups	1.288	10	0.129		
	Total	11.252	14			
Prorionaldehyde_Canadian	Between Groups	7.048	4	1.762	1.743	0.217
	Within Groups	10.106	10	1.011		
	Total	17.154	14			
Butyraldehyde_ISO	Between Groups	3.881	4	0.970	13.454	0.000
	Within Groups	0.721	10	0.072		
	Total	4.602	14			
Butyraldehyde_Canadian	Between Groups	3.405	4	0.851	1.564	0.257
	Within Groups	5.441	10	0.544		
	Total	8.845	14			

Table 3. Impact of smoking conditions on the produced compound

**Independent Samples Test**										
		Levene's Test for Equality of Variances		t-test for Equality of Means						
		F	Sig.	t	df	Sig. (2-tailed)	Mean Difference	Std. Error Difference	95% Confidence Interval of the Difference	
									Lower	Upper
Acetaldehyde	Equal variances assumed	3.128	0.088	-2.327	28	0.027	-8.24000	3.54147	-15.49437	-0.98563
	Equal variances not assumed			-2.327	24.395	0.029	-8.24000	3.54147	-15.54298	-0.93702
Propionaldehyde	Equal variances assumed	0.802	0.378	-6.638	28	0.000	-2.441383806	0.367787459	-3.194762263	-1.688005348
	Equal variances not assumed			-6.638	26.842	0.000	-2.441383806	0.367787459	-3.196229815	-1.686537797

Butyraldehyde	Equal variances assumed	0.973	0.332	-4.205	28	0.000	-1.064109232	0.253054756	-1.582468401	-0.545750063
	Equal variances not assumed			-4.205	25.465	0.000	-1.064109232	0.253054756	-1.584803069	-0.543415395

Table 4. Statistics for each produced compound

**Group Statistics**					
**Compound**	**Smoking Condition**	**N**	**Mean**	**Std. Deviation**	**Std. Error Mean**
Acetaldehyde	ISO	15	45.4787	7.60952	1.96477
	Canadian	15	53.7187	11.41163	2.94647
Propionaldehyde	ISO	15	4.38819309	0.896519762	0.231480407
	Canadian	15	6.82957689	1.106917586	0.285804892
Butyraldehyde	ISO	15	3.81655261	0.573362-19	0.148041437
	Canadian	15	4.88066184	0.794862653	0.205232654

Table 5. Impact of smoking conditions on the produced acetaldehyde for each brand

**Independent Samples Test**										
		Levene's Test for Equality of Variances		t-test for Equality of Means						
		F	Sig.	t	df	Sig. (2-tailed)	Mean Difference	Std. Error Difference	95% Confidence Interval of the Difference	
									Lower	Upper
Acetaldehyde_IQOS	Equal variances assumed	6.083	0.069	-3.637	4	0.022	-18.46333	5.07648	-32.55790	-4.36877
	Equal variances not assumed			-3.637	2.358	0.053	-18.46333	5.07648	-37.41667	0,49000
Acetaldehyde_LIL	Equal variances assumed	2.976	0.160	-2.501	4	0.067	-17.10333	6.83823	-36.08930	1.88264
	Equal variances not assumed			-2.501	2.836	0.092	-17.10333	6.83823	-39.59499	5.38832
Acetaldehyde_GLO	Equal variances assumed	0.001	0.974	1.054	4	0.352	1.74667	1.65796	-2.85656	6.34989
	Equal variances not assumed			1.054	3.962	0.352	1.74667	1.65796	-2.87402	6.36736
Acetaldehyde_ILU	Equal variances assumed	0.251	0.643	-0.306	4	0.775	-2.22667	7.27929	-22.43721	17.98388
	Equal variances not assumed			-0.306	3.914	0.775	-2.22667	7.27929	-22.61454	18.16120
Acetaldehyde_PULZE	Equal variances assumed	1.735	0.258	-1.332	4	0.254	-5.15333	3.86776	-15.89196	5.58529
	Equal variances not assumed			-1.332	3.262	0.268	-5.15333	3.86776	-16.92108	6.61441

Table 6. Statistics for acetaldehyde for each brand

**Group Statistics**					
**Compound**	**Smoking Condition**	**N**	**Mean**	**Std. Deviation**	**Std. Error Mean**
Acetaldehyde _IQOS	ISO	3	44.0100	2.52929	1.46029
	Canadian	3	62.4733	8.42108	4.86191
Acetaldehyde _LIL	ISO	3	39.6333	5.02060	2.89865
	Canadian	3	56.7367	10.72743	6.19348
Acetaldehyde _GLO	ISO	3	38.2833	1.92858	1.11347
	Canadian	3	36.5367	2.12768	1.22842

Acetaldehyde _ILU	ISO	3	53.1567	8.22606	4.74932
	Canadian	3	55.3833	9.55490	5.51652
Acetaldehyde _PULZE	ISO	3	52.3100	3.43044	1.98056
	Canadian	3	57.4633	5.75420	3.32219

Table 7. Impact of smoking conditions on the produced propionaldehyde for each brand

Independent Samples Test										
		Levene's Test for Equality of Variances		t-test for Equality of Means						
		F	Sig.	t	df	Sig. (2-tailed)	Mean Difference	Std. Error Difference	95% Confidence Interval of the Difference	
									Lower	Upper
Propionaldehyde_IQOS	Equal variances assumed	4.996	0.089	-9.940	4	0.001	-3.381692478	0.340217195	-4.326286843	-2.437098112
	Equal variances not assumed			-9.940	2.598	0.004	-3.381692478	0.340217195	-4.565611015	-2.197773940
Propionaldehyde _LIL	Equal variances assumed	0.558	0.496	-6.522	4	0.003	-3.075039812	0.471483621	-4.384088204	-1.765991420
	Equal variances not assumed			-6.522	3.569	0.004	-3.075039812	0.471483621	-4.448787497	-1.701292127
Propionaldehyde _GLO	Equal variances assumed	4.737	0.095	-2.387	4	0.075	-2.319893795	0.971876199	-5.018254710	0.378467120
	Equal variances not assumed			-2.387	2.082	0.135	-2.319893795	0.971876199	-6.347177509	1.707389919
Propionaldehyde _ILU	Equal variances assumed	2.582	0.183	-2.049	4	0.110	-1.413124305	0.689610216	-3.327789212	0.501540603
	Equal variances not assumed			-2.049	2.966	0.134	-1.413124305	0.689610216	-3.622174392	0.795925783
Propionaldehyde_PULZE	Equal variances assumed	2.195	0.213	-5.375	4	0.006	-2.017168639	0.375260456	-3.059058694	-0.975278583
	Equal variances not assumed			-5.375	2.314	0.024	-2.017168639	0.375260456	-3.439170974	-0.595166303

Table 8. Statistics for propionaldehyde for each brand

Group Statistics					
**Compound**	**Smoking Condition**	**N**	**Mean**	**Std. Deviation**	**Std. Error Mean**
Propionaldehyde_IQOS	ISO	3	4.18457576	0.214678498	0.123944689
	Canadian	3	7.56626824	0.548777151	0.316836636
Propionaldehyde_LIL	ISO	3	4.12447439	0.466505058	0.269336821
	Canadian	3	7.19951420	0.670271173	0.386981242
Propionaldehyde_GLO	ISO	3	3.54435161	0.238985932	0.137978592
	Canadian	3	5.86424540	1.666288019	0.962031836
Propionaldehyde_ILU	ISO	3	5.94902934	0.540456094	0.312032471
	Canadian	3	7.36215364	1.065173206	0.614978037
Propionaldehyde_PULZE	ISO	3	4.13853435	0.175755404	0.101472430
	Canadian	3	6.15570299	0.625756556	0.361280716

Table 9. Impact of smoking conditions on the produced butyraldehyde for each brand

Independent Samples Test									
	Levene's Test for Equality of Variances		t-test for Equality of Means						
	F	Sig.	t	df	Sig. (2-tailed)	Mean Difference	Std. Error Difference	95% Confidence Interval of the Difference	

									Lower	Upper
Butyraldehyde _IQOS	Equal variances assumed	2.956	0.161	-4.018	4	0.016	-1.577785856	0.392724665	-2.668164330	-0.487407382
	Equal variances not assumed			-4.018	2.151	0.050	-1.577785856	0.392724665	-3.158878565	0.003306853
Butyraldehyde _LIL	Equal variances assumed	3.934	0.118	-4.019	4	0.016	-1.318449248	0.328037397	-2.229227073	-0.407671423
	Equal variances not assumed			-4.019	2.048	0.054	-1.318449248	0.328037397	-2.698744961	0.061846466
Butyraldehyde _GLO	Equal variances assumed	11.357	0.028	-1.771	4	0.151	-0.868140594	0.490305539	-2.229447006	0.493165819
	Equal variances not assumed			-1.771	2.067	0.215	-0.868140594	0.490305539	-2.913473162	1.177191975
Butyraldehyde _ILU	Equal variances assumed	0.601	0.481	-0.560	4	0.605	-0.313660512	0.560254792	-1.869177186	1.241856162
	Equal variances not assumed			-0.560	3.181	0.613	-0.313660512	0.560254792	-2.040652995	1.413331972
Butyraldehyde _PULZE	Equal variances assumed	1.573	0.2278	-2.706	4	0.054	-1.242509951	0.459178399	-2.517393570	0.032373668
	Equal variances not assumed			-2.706	2.695	0.083	-1.242509951	0.459178399	-2.802068219	0.317048317

Table 10. Statistics for butyraldehyde for each brand

Group Statistics

**Compound**	**Smoking Condition**	**N**	**Mean**	**Std. Deviation**	**Std. Error Mean**
Butyraldehyde_IQOS	ISO	3	3.93671662	0.129780286	0.074928683
	Canadian	3	5.51450248	0.667723794	0.385510512
Butyraldehyde _LIL	ISO	3	3.43942329	0.061776994	0.035666964
	Canadian	3	4.75787254	0.564808998	0.326092627
Butyraldehyde_GLO	ISO	3	3.19501093	0.109093565	0.062985199
	Canadian	3	4.06315153	0.842197814	0.486243134
Butyraldehyde_ILU	ISO	3	4.68292702	0.481549701	0.278022849
	Canadian	3	4.99658753	0.842476220	0.486403872
Butyraldehyde_PULZE	ISO	3	3.82868518	0.310095991	0.179034004
	Canadian	3	5.07119513	0.732376190	0.422837590

© 2026 Zervas E. et al.
